# Impact of Residential Segregation on Healthcare Utilization and Perceived Quality of Care Among Informal Caregivers in the United States

**DOI:** 10.1007/s40615-024-02018-9

**Published:** 2024-05-17

**Authors:** Hyunmin Kim, Asos Mahmood, Satish Kedia, Deborah O. Ogunsanmi, Sadikshya Sharma, David K. Wyant

**Affiliations:** 1 College of Nursing and Health Professions, School of Health Professions, The University of Southern Mississippi, Hattiesburg, MS USA; 2 Center for Health System Improvement, College of Medicine, University of Tennessee Health Science Center, 956 Court Ave Avenue, Ste D222A, Memphis, TN 38103 USA; 3 Department of Medicine-General Internal Medicine, College of Medicine, University of Tennessee Health Science Center, Memphis, TN USA; 4 Division of Social and Behavioral Sciences, School of Public Health, The University of Memphis, Memphis, TN USA; 5 Tennessee Population Health Consortium and Institute for Health Outcomes and Policy Research, University of Tennessee Health Science Center, Memphis, TN USA; 6 Jack C. Massey College of Business, Frist College of Medicine, Belmont University, Nashville, TN USA

**Keywords:** Residential segregation, Informal caregivers, Race, Healthcare utilization, Quality of care, Healthcare disparities

## Abstract

This study aimed to investigate the impact of racial residential segregation on healthcare utilization and perceived quality of care among informal caregivers in the US. It further assessed potential variations in the estimated impact across caregivers’ race and socioeconomic status. We used data from the Health Information National Trends Survey Data Linkage Project (fielded in 2020) for a sample of 583 self-identified informal caregivers in the US. Fitting a series of regression models with the maximum likelihood estimation, we computed the beta coefficients (*β*) of interest and their associated Wald 95% confidence limits (CI). Caregivers who resided in areas with higher segregation, compared to those living in lower segregated areas, were less likely to visit a healthcare professional [*β* =  − 2.08; Wald 95%CI − 2.093, − 2.067] (moderate); [*β* =  − 2.53; Wald 95%CI − 2.549, − 2.523] (high)]. Further, caregivers residing in moderate [*β* =  − 0.766; Wald 95%CI − 0.770, − 0.761] and high [*β* =  − 0.936; Wald 95%CI − 0.941, − 0.932] segregation regions were less likely to perceive a better quality of care compared to those located in low segregation areas. Moreover, as segregation level increased, Black caregivers were less likely to see a health professional, less frequently used healthcare services, and had poorer perceived healthcare quality when compared to Whites. Our findings indicate that higher residential segregation is associated with lower healthcare utilization, such as visiting a healthcare professional, and poorer perceived healthcare quality among informal caregivers. Given the essential role of informal caregivers in the current healthcare system, it is vital to investigate and address challenges associated with access to and quality of essential healthcare services to improve caregivers’ health and well-being, specifically for caregivers of minority backgrounds.

## Introduction

Informal caregivers provide important and unpaid voluntary care to family members or other loved ones who need assistance [1, 2]. With the rapidly aging population and increasing prevalence of chronic medical conditions in the United States (US) and worldwide, the role of caregivers, both formal and informal, has become paramount [3]. Currently, there are approximately 53 million self-identified informal caregivers in the US [4]. These caregivers are reportedly providing care to mainly middle-aged and older adults (those aged ≥ 50 years) with an estimated 41.8 million care recipients in the US [3, 5].

Due to the burden associated with caregiving responsibilities, caregivers often experience adverse physical and mental health issues and an overall decline in quality of life and well-being due to challenges such as longstanding insomnia, exhaustion, fatigue, and a poor diet [6]. Caregivers are also subject to adverse mental health and psychological issues, including stress, anxiety, and depressive symptoms [7]. One previous study found that family caregivers of patients with lung cancer had a substantially lower quality of physical and mental health than the general population [8]. In addition, the association between informal caregiving responsibilities and self-reported poor health strengthened with the amount and duration of caregiving duties [9]. Further, evidence indicates that there is an association between informal caregiver burden and increased risk of cardiovascular diseases, including hypertension [10, 11], heart disease [12–14], and higher mortality [15]. Another study reported that caregivers who provided care to their disabled spouses had a 63% increased risk of mortality compared to non-caregivers at a 4-year follow-up in the US [16].

Furthermore, informal caregivers are prone to higher rates of anxiety, depression, fear, and uncertainty associated with their caregiving responsibilities, affecting their well-being and overall quality of life [17–23]. Although the association of caregiving burden with psychological distress varies among different caregiver sociodemographic groups, it is still substantially stronger among informal caregivers compared to the general population [24]. For example, a prior meta-analysis found large differences in depression, stress, and general subjective well-being levels between informal caregivers and non-caregivers [25].

Access to quality healthcare is paramount for informal caregivers to sustain their health and well-being while providing care to their loved ones. However, caregiving responsibilities limit their time availability and interfere with scheduling medical appointments, thus prioritizing the needs of their care recipients over their own well-being [26, 27]. A prior study conducted among lung cancer patients’ family caregivers in the US found that caregivers with clinically meaningful psychological distress did not utilize support services [28]. Another study among informal caregivers of advanced cancer patients found that less than 50% of caregivers with a current psychiatric disorder used mental health services [29]. Other studies also report that informal caregivers in general are less likely to utilize needed healthcare services [30–32]. However, current evidence regarding healthcare utilization among informal caregivers is not consistent. For example, a study conducted among spousal caregivers of persons with dementia reported that caregivers had a higher number of emergency room (ER) visits [33]. Others have found no significant differences in hospitalizations among informal caregivers compared to non-caregivers [34–36], while a few others indicate higher rates of outpatient visits among caregivers [37–39].

Currently, little is known about factors influencing healthcare access and utilization and the quality of care received among informal caregivers in the US. In particular, we do not know much about the association between residential segregation and healthcare utilization and quality of care among informal caregivers. Residential segregation is a form of institutional racism that involves physically separating different racial groups, particularly African Americans [40]. The discriminatory practices and policies promoting segregation have been abolished and ruled illegal for decades in the US. However, their long-term adverse consequences persist up to the present time [41]. While extreme levels of segregation are experienced by African Americans, many immigrant groups in the US have historically faced some degree of residential segregation [40, 42]. Residential segregation plays a crucial role in creating disparities in access to and utilization of needed healthcare services [43]. It potentially affects caregivers’ health due to its impact on shaping socioeconomic status (SES), access to education, employment opportunities, transportation, nutritious food, healthcare information, and other factors resulting in pronounced health and economic disparities [40, 44]. Prior studies in the field have found links between racial residential segregation and hypertension [45], obesity [46], cancer [47], cardiovascular diseases [45, 48, 49], COVID-19 infection [50], infant and maternal mortality [51], childhood asthma [52], and many other health conditions [53]. The current study builds upon the gaps in the literature and investigates associations of residential segregation with healthcare service utilization and perceived healthcare quality among informal caregivers in the US. We hypothesize that informal caregivers residing in locations with higher residential segregation (Black vs. White) are less likely to utilize healthcare services and less likely to have a good quality of care. We further hypothesize that adverse impacts of higher residential segregation on healthcare access and quality are more prominent among caregivers of minority backgrounds and those of lower socioeconomic status.

## Methods

### Data and Study Sample

This study used the Health Information National Trends Survey (HINTS) Data Linkage Project 2020 (HDLP) as a linked, combined, dataset of HINTS 5 (Cycle 4) and other multiple reliable data sources (including the US Census, the Agency for Healthcare Research and Quality (AHRQ), and the US Department of Agriculture) in the US [54, 55]. HINTS is a national cross-sectional survey, conducted among civilian, non-institutionalized US adults aged 18 years and older, and administered by the National Cancer Institute. Restricted HINTS data underlying the current study were linked at the US county level with various access contextual measures such as social and economic factors (i.e., segregation index, income inequality), physical environment (i.e., air quality), and built environment (i.e., fitness centers per 100,000 people).

The survey questionnaire included items related to uncompensated caregiving responsibility and caregivers, who were defined as “Participants who are currently caring for, or making healthcare decisions for, someone with a medical, behavioral, disability, or other condition.” Survey respondents were asked whether they have caregiving responsibilities (yes vs. no). Participants with an affirmative response were further asked, “Please check all conditions for which you have provided care for this person,” with the ability to mark all applicable caregiving conditions, including caring for individuals with cancer, Alzheimer’s disease, confusion, dementia, forgetfulness, orthopedic/musculoskeletal issues, mental health/behavioral/substance abuse issues, chronic conditions, neurological/developmental issues, acute conditions, and aging/aging-related health issues. Among 3865 survey respondents, our study’s analytical sample resulted in 583 self-identified informal caregivers after excluding the non-caregivers (*n* = 2975), as well as missing values and incorrect observational information (*n* = 307) from the sample dataset.

### Measures and Main Variables

The dependent variables of the current study assessed (1) whether a caregiver had a healthcare visit in the past year; (2) the frequency of healthcare visits in the past year; and (3) the quality of care received. These outcome measures were created based on the survey questionnaires, “In the past 12 months, not counting times you went to an emergency room, how many times did you go to a doctor, nurse, or other health professional to get care for yourself?” and “Overall, how would you rate the quality of healthcare you received in the past 12 months?” Concerning the first question, survey respondents were able to answer with the following response categories: “none,” “1 time,” “2 times,” “3 times,” “4 times,” “5–9 times,” and “10 or more times.” To assess whether caregivers used healthcare in the past year, we utilized the first question to create a binary variable (i.e., none corresponding to “no” and 1 or more times corresponding to “yes”). Regarding the question on quality healthcare received, respondents answered with the following response categories: “excellent,” “very good,” “good,” “fair,” and “poor.” We re-categorized these response options into “excellent/very good,” “good,” and “fair or poor.”

**Fig. 1:**
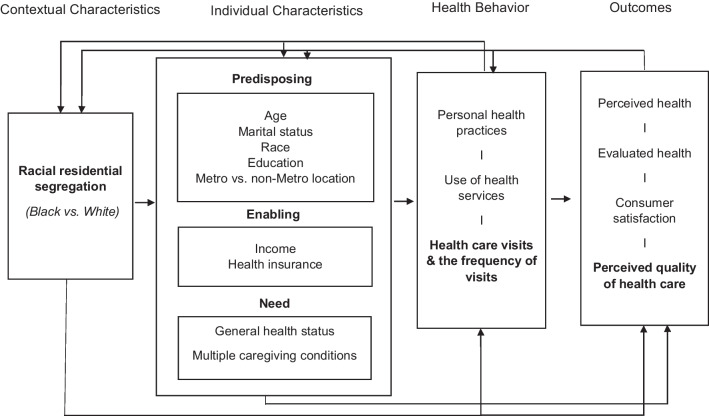
A conceptual framework adopted from Andersen’s Behavioral Model of Health Services Utilization to examine relationships of racial residential segregation (Black vs. White) with healthcare utilization, and perceived quality of health care among informal caregivers in the USA

The main independent variable was levels of segregation, measured by the segregation index of dissimilarity (X) (i.e., the degree of residential segregation between Black and White county residents), with higher values indicating greater residential segregation between Black and White county residents. This variable was treated as both “continuous,” scores 0–100, and “categorical” by recoding it into three levels (“low” if 0 < X ≤ 30; “moderate” if 30 < X ≤ 60; and “high” 60 < X ≤ 100). The source of this segregation index is the County Health Rankings from the AHRQ Social Determinants of Health 2019 Dataset. Guided by Andersen and Newman’s model of healthcare utilization [56, 57], the study included three groups of predisposing, enabling, and need-for-care factors as potential predictors for healthcare utilization and perceived quality of healthcare (see Fig. 1). Predisposing factors consisted of demographic characteristics (age, race, gender, education, marital status, and metro vs. non-metro location status). Age was included as a categorical variable in the model (“18–34 years,” “35–39 years,” “40–44 years,” and “45 + years”). Black (or African American) race and female gender were both included as binary variables. Education was categorized into four levels: “less than high school,” “high school graduate,” “some college,” and “college graduate or more.” Marital status was grouped into three subcategories “single or never been married,” “married/living as married,” and “divorced/widowed/separated.” The metropolitan residential location of caregivers was included as a binary variable (metro vs. non-metro). Assigning this residential location of caregivers was dependent on the 2013 US Department of Agriculture Rural–Urban Continuum Codes [58].

Enabling factors consisted of income and health insurance coverage status. Income was treated as a categorical variable (“ < $35,000,” “$35,000 to < $100,000,” and “ ≥ $100,000”), and health insurance coverage was included as a binary variable. The incorporated need-for-care measures were self-rated general health status (“excellent/very good,” “good,” and “fair or poor”), and a series of caregiving conditions, or responsibilities, that the caregivers reported to provide care for.

### Statistical Analysis

Given that about 33% of US counties were represented in the HDLP data, we incorporated HINTS weights in our statistical analyses to represent the US informal caregiver population. We estimated descriptive statistics for the characteristics of informal caregivers and the differences by segregation level (the frequency and weighted percentages of each variable of interest). To ensure that our sample was reflective of the population and correct for sampling-related biases, we incorporated replicate weights that were computed using the delete one Jackknife replication method. Associations between levels of segregation and healthcare utilization among informal caregivers (*n* = 583) were assessed using Firth’s penalized logistic regression analyses to resolve issues related to using a general logistic regression model, i.e., a quasi-separation or non-convergence on the estimate [59, 60]. In this instance, given that the dependent variable “healthcare visits” was dichotomous, where most response categories were “yes,” it was plausible that the maximum likelihood estimation did not exist when using a general logistic regression approach. Next, among those informal caregivers who visited a healthcare professional in the past year (*n* = 510), given that the dependent variable “frequency of healthcare visits” was ordinal, we examined the relationship between the frequency of healthcare visits attributed to segregation levels by analyzing a proportional odds model.

In sensitivity analysis, we also employed different regression models such as Poisson and negative binomial regression models; however, those models did not fit into our data. The model assumes that although the intercepts’ estimates are different, the parameters should be the same. We performed several tests to investigate and select the best-fit model. Specifically, we conducted a *F-*test to compare our model to the model with no predictors, with which results showed a significant result (*P* < 0.001). Further, the results of the Akaike Information Criterion (AIC) as an indicator of the goodness of fit of a statistical model showed that the chosen adjusted models on the three outcome variables were the better fit models compared with the ones without covariates (AIC 11783275 vs.16640784 [Number of healthcare visits in the past 12 months]; AIC 32314358 vs. 40207523 [Healthcare visits in the past 12 months]; AIC 54247742 vs. 63713306 [Quality of healthcare received]).

Subsequently, we examined associations between perceived quality of care received and segregation levels among informal caregivers who used healthcare at least once during the past year by fitting an ordinal multinomial logistic regression model. Further, we investigated whether, and to what extent, the associations differed by race, and socioeconomic status (assessed across education and income levels) using the interaction terms (“segregation × race,” “segregation × income,” and “segregation × education”). Moreover, we conducted a subgroup analysis by residential location (metropolitan vs. non-metropolitan) to assess if there exist any significant differences in healthcare utilization and perceived quality of care attributed to segregation between informal caregivers across metropolitan and non-metropolitan areas. Specifically, we analyzed a stratified regression model by metropolitan status as binary, in that the relationship between segregation and healthcare utilization and perceived quality of care was examined in the metropolitan and non-metropolitan areas separately, given the caregivers’ metro vs. non-metro resident status. All statistical analyses were performed using the SAS (version 9.4) statistical software program (SAS Institute Inc., Cary, NC, USA ©2014), and the significance threshold was set at *P*-value < 0.05.

## Results

The majority (61.06%) of the informal caregivers was women, aged 45 years and older (65.30%), married or living as married (65.89%), and living in metropolitan areas (84.31%) (see Table 1). Nearly all (92.62%) caregivers had health insurance coverage, and 87.77% went to see a healthcare professional at least once in the past 12 months. A small proportion of informal caregivers (13.09%) self-identified as Black and 27.69% had an annual income of ≥ $100,000. Close to 45.70% had some college education, and 28.22% were college graduates or had more education. Among those who used healthcare in the past year, 65.04% rated the quality of care received as “excellent/very good,” while 8.20% reported fair or poor quality of care received. Approximately half (45.33%) of caregivers had multiple responsibilities in providing care to individuals with various health conditions, while slightly more than 40% were providing care for individuals with only one condition. More caregiving men than women reported residing in areas with a higher level of segregation. Caregivers who were married or living as married, and those with higher incomes (≥ $100,000), tended to live in areas with a lower level of segregation, while singles and those with lower incomes (< $35,000) were more likely to reside in areas with a higher level of segregation (see Table 1). Furthermore, caregivers residing in metropolitan locations tended to live in areas with a higher level of segregation. Caregivers of patients with acute conditions and aging-related health issues were more likely to live in areas with higher segregation, while those who cared for patients with Alzheimer’s disease, confusion, dementia, forgetfulness, chronic conditions, and neurological/developmental issues tended to reside in areas with lower segregation.

**Table 1:** Characteristics of informal caregivers by segregation level (HINTS Data Linkage Project 2020, the United States)

Characteristic	Caregivers (*N* = 583)		Segregation index^b,c^		
			High (*n* = 120)	Moderate (*n* = 421)	Low (*n* = 30)
	*n*^a^	Weighted % (SE)	Weighted % (SE)	Weighted % (SE)	Weighted % (SE)
Age groups (in years)					
18 - 34	57	16.16 (3.10)	14.52 (6.05)	16.85 (3.92)	25.03 (16.59)
35 - 39	46	9.61 (1.62)	7.06 (2.49)	11.34 (2.22)	–
40 - 44	43	8.93 (1.61)	6.85 (2.70)	10.13 (2.01)	1.33 (1.39)
45 +	420	65.30 (3.52)	71.57 (5.58)	61.68 (4.71)	73.64 (16.55)
Not reported	17	–	–	–	–
Female	356	61.06 (2.71)	47.50 (6.51)	65.50 (3.34)	69.35 (13.47)
Black race	90	13.09 (2.43)	12.41 (3.54)	14.01 (2.83)	11.44 (6.47)
Marital status					
Married/living as married	364	65.89 (3.25)	49.94 (6.75)	68.22 (3.85)	86.62 (7.06)
Divorced/widowed/separated	128	12.65 (1.66)	9.53 (2.63)	14.39 (2.51)	7.86 (5.06)
Single (never been married)	76	21.46 (3.05)	40.53 (7.19)	17.39 (3.24)	5.52 (4.16)
Not reported	15	–	–	–	–
Income (Y)					
Y < $35,000	181	32.65 (3.54)	45.64 (7.09)	28.78 (3.24)	19.50 (10.49)
$35,000 ≤ Y < $100,000	238	39.66 (2.94)	33.59 (6.97)	42.63 (3.80)	38.27 (16.92)
Y ≥ $100,000	161	27.69 (2.71)	20.77 (4.54)	28.59 (3.31)	42.23 (18.92)
Not reported	3	–	–	–	–
Non-metropolitan residential location	65	15.69 (2.52)	12.17 (5.78)	11.73 (3.02)	41.43 (17.62)
Education					
Less than high school	34	5.31 (1.22)	5.44 (2.98)	4.48 (1.35)	18.42 (17.15)
High school graduate	96	20.77 (2.93)	21.94 (8.21)	20.53 (3.67)	16.02 (10.18)
Some college	179	45.70 (3.01)	42.72 (6.61)	46.15 (3.71)	41.75 (18.41)
College graduate or more	256	28.22 (2.27)	29.90 (5.32)	28.84 (2.88)	23.81 (10.12)
Not reported	18	–	–	–	–
Health insurance	526	92.62 (2.32)	98.12 (1.21)	90.79 (2.30)	100.00 ( -)
General health status					
Excellent/very good	246	39.59 (2.39)	42.69 (7.52)	40.35 (2.64)	21.97 (11.74)
Good	224	39.98 (2.95)	37.89 (7.55)	40.16 (3.29)	51.97 (16.25)
Fair or poor	109	20.43 (3.06)	19.42 (5.59)	19.49 (3.39)	26.06 (15.68)
Not reported	4	–	–	–	–
Caregiving conditions/responsibilities					
Cancer	14	1.46 (0.44)	–	1.66 (0.47)	–
Alzheimer’s, confusion, dementia, forgetfulness	26	2.91 (0.69)	2.70 (1.76)	2.56 (0.86)	9.83 (5.52)
Orthopedic/musculoskeletal issues	14	2.95 (1.06)	–	3.67 (1.41)	–
Mental health/behavioral/substance abuse issues	59	13.41 (2.65)	2.53 (1.22)	16.30 (3.04)	3.77 (3.65)
Chronic conditions	42	9.20 (2.20)	4.28 (1.77)	10.43 (2.90)	15.96 (16.09)
Neurological/developmental issues	29	4.97 (1.14)	3.49 (2.58)	5.44 (1.32)	8.52 (8.56)
Acute conditions	12	3.74 (1.40)	8.56 (5.29)	2.64 (1.21)	–
Aging/aging-related health issues	26	4.95 (1.51)	6.55 (3.42)	4.88 (1.64)	–
Multiple caregiving conditions	284	45.33 (3.43)	55.86 (7.04)	42.24 (4.09)	53.35 (16.62)
Not sure	41	5.34 (1.07)	8.46 (2.90)	4.79 (1.12)	2.14 (2.28)
Other	36	5.74 (1.15)	7.57 (2.89)	5.39 (1.38)	6.43 (4.91)
Healthcare visits in the past 12 months	510	87.77 (2.29)	98.56 (1.44)	97.22 (1.46)	98.55 (1.54)
Number of healthcare visits in the past 12 months					
None	63	12.22 (2.29)	13.75 (3.74)	12.77 (3.02)	1.44 (1.54)
1 time	68	13.50 (2.28)	10.39 (3.56)	12.99 (2.71)	31.22 (15.85)
2 times	111	18.51 (3.47)	20.25 (5.71)	18.48 (4.58)	3.38 (3.66)
3 times	96	15.17 (2.08)	9.78 (3.80)	16.90 (2.57)	21.12 (11.81)
4 times	71	10.82 (1.92)	10.56 (5.48)	10.67 (2.06)	10.16 (5.30)
5 ~ 9 times	102	18.24 (3.09)	16.45 (5.54)	18.64 (2.91)	8.93 (4.58)
10 or more times	62	11.54 (2.37)	17.92 (6.13)	9.55 (2.33)	23.75 (18.99)
Not reported/error	10	–	–	–	–
Quality of healthcare received^d^					
Excellent/very good	361	65.04 (3.12)	61.50 (8.09)	62.43 (3.90)	78.77 (9.00)
Good	119	26.74 (3.46)	27.34 (8.04)	25.84 (4.26)	16.64 (7.98)
Fair or poor	35	8.22 (2.00)	11.16 (4.40)	11.73 (2.97)	4.59 (3.27)
Not reported/error/no healthcare visits in past 12 months	68	–	–	–	–

*HINTS*, Health Information National Trends Survey

^a^Unweighted sample frequencies

^b^Segregation index (X) was categorized into three levels: “low” if 0 < X ≤ 30; “moderate” if 30 < X ≤ 60; and “high” if 60 < X ≤ 100

^c^The frequencies for subcategories are not reported in compliance with the data use agreement with the National Cancer Institute. These subcategories had a very small cell count and could pose a risk for some respondent identification

^d^Informal caregivers with at least one healthcare visit in the past 12 months

Table 2 presents findings from regression analyses. Compared to caregivers residing in areas with low segregation, those who lived in areas with higher segregation were less likely to visit a healthcare professional [*β* =  − 2.08; Wald 95%CI: − 2.093, − 2.067] (moderate); [*β* =  − 2.536; Wald 95%CI: − 2.549, − 2.523] (high)]. That means, when compared to informal caregivers residing in regions with lower levels of segregation, those living in areas with moderate and high segregation had a 208 and 253 percentage point (PP) lower probability of visiting a healthcare professional, respectively. Similarly, among caregivers who reported healthcare professional visits during the past year, residing in areas with a higher level of segregation was associated with a lower frequency of visits [*β* =  − 0.470; Wald 95%CI: − 0.473, − 0.466] (moderate); [*β* =  − 0.148; Wald 95%CI: − 0.151, − 0.144] (high)] and poorer perceived healthcare quality [*β* =  − 0.766; Wald 95%CI: − 0.770, − 0.761] (moderate); [*β* =  − 0.936; Wald 95%CI: − 0.941, − 0.932] (high)].

**Table 2:** Generalized linear models for examining healthcare visits and frequency, and perceived quality of healthcare among informal caregivers (HINTS Data Linkage Project 2020, the United States)

All caregivers (*n* = 583)			Caregivers who visited a health professional in past year (*n* = 510)			
Characteristics	Healthcare visit		Frequency of healthcare visits		Quality of healthcare	
	Estimate	Wald 95% CL	Estimate	Wald 95% CL	Estimate	Wald 95% CL
Segregation (ref.: low)						
Moderate	− 2.080	[− 2.093, − 2.067]	− 0.470	[− 0.473, − 0.466]	− 0.766	[− 0.770, − 0.761]
High	− 2.536	[− 2.549, − 2.523]	− 0.148	[− 0.151, − 0.144]	− 0.936	[− 0.941, − 0.932]
Age groups (in years) (ref.: 45 +)						
18–34	− 0.251	[− 0.254, − 0.248]	0.253	[0.251, 0.255]	− 0.842	[− 0.845, − 0.840]
35–39	− 0.267	[− 0.270, − 0.263]	− 0.302	[− 0.304, − 0.299]	− 0.538	[− 0.540, − 0.535]
40–44	− 1.204	[− 1.207, − 1.200]	− 0.219	[− 0.222, − 0.217]	− 0.678	[− 0.681, − 0.675]
Female	− 0.610	[− 0.613, − 0.608]	0.175	[0.174, 0.177]	0.210	[0.208, 0.212]
Black race (ref: non-Black)	− 0.375	[− 0.378, − 0.372]	− 0.481	[− 0.483, − 0.478]	0.928	[0.925, 0.931]
Marital status (ref.: divorced/widowed/separated)						
Married/living as married	0.032	[0.029, 0.036]	0.348	[0.346, 0.350]	− 0.229	[0.473, 0.479]
Single (never been married)	0.391	[0.387, 0.395]	0.854	[0.852, 0.857]	0.476	[− 0.232, − 0.226]
Income (Y) (ref.: Y ≥ $100,000)						
Y < $35,000	0.457	[0.453, 0.461]	0.159	[0.157, 0.162]	− 0.769	[− 0.772, − 0.766]
$35,000 ≤ Y < $100,000	0.199	[0.196, 0.202]	0.004	[0.002, 0.006]	0.006	[0.004, 0.009]
Metropolitan residential location	− 0.641	[− 0.645, − 0.637]	0.636	[0.634, 0.638]	0.086	[0.083, 0.088]
Education (ref.: less than high school)						
High school graduate	− 0.752	[− 0.758, − 0.747]	1.741	[1.738, 1.745]	− 0.597	[− 0.601, − 0.593]
Some college	− 0.485	[− 0.490, − 0.479]	1.317	[1.313, 1.320]	− 1.168	[− 1.172, − 1.164]
College graduate or more	0.255	[0.250, 0.261]	1.604	[1.600, 1.607]	− 1.151	[− 1.156, − 1.147]
Health Insurance	0.930	[0.926, 0.934]	− 0.378	[− 0.381, − 0.376]	0.410	[0.407, 0.413]
General health status (ref.: fair or poor)						
Excellent/very good	− 0.836	[− 0.839, − 0.832]	− 1.941	[− 1.943, − 1.939]	2.090	[2.088, 2.093]
Good	− 0.834	[− 0.838, − 0.831]	− 0.446	[− 0.448, − 0.444]	1.244	[1.242, 1.246]
Multiple caregiving conditions	− 0.716	[− 0.719, − 0.713]	0.780	[0.778, 0.781]	0.290	[0.289, 0.292]

Abbreviations CL, Confidence limits; Ref, reference

Further, we explored racial and socioeconomic differences in the association between residential segregation and healthcare utilization and the quality of care received among informal caregivers (see Table 3). Specifically, we examined the interactive effects of race (“White” and “Black”), income, and education with segregation levels on a continuous scale. Compared to their White counterparts, as segregation level increased, Black caregivers were less likely to have a healthcare professional visit [*β* =  − 0.0011; Wald 95%CI: − 0.0012, − 0.0011], used healthcare less frequently [*β* =  − 0.0290; Wald 95%CI: − 0.0291, − 0.0289], and had poorer perceived quality of healthcare [*β* =  − 0.0158; Wald 95%CI: − 0.0159, − 0.0158]. Moreover, albeit with some differences, overall, it appeared that as segregation levels increased, informal caregivers with lower incomes were less likely to see a healthcare professional (see Table 3). With regard to education in the association between residential segregation and healthcare utilization and quality of care received, no conclusive results were found with consistency. For instance, compared with those with a college degree or more education, informal caregivers with lower education (“less than high school”) were less likely to visit a healthcare professional and use healthcare less frequently, but were more likely to perceive a higher quality of care (see Table 3).

**Table 3:** Interactive effects of race and socioeconomic status, assessed through income and education level, in the relationships between segregation and healthcare utilization and perceived quality of care among informal caregivers (HINTS Data Linkage Project 2020, the United States)

Interactions	Healthcare visit		Frequency of healthcare visits		Quality of healthcare received	
	Estimate	Wald 95% CL	Estimate	Wald 95% CL	Estimate	Wald 95% CL
Segregation × race (Black)	− 0.0011	[− 0.0012, − 0.0011]	− 0.0290	[− 0.0291, − 0.0289]	− 0.0158	[− 0.0159, − 0.0158]
Segregation × income (< $35,000)	− 0.0206	[− 0.0207, − 0.0206]	0.0133	[0.0133, 0.0134]	0.0106	[0.0105, 0.0106]
Segregation × Income ($35,000 ≤ Y < $100,000)	− 0.0119	[− 0.0119, − 0.0118]	0.0016	[0.0016, 0.0016]	0.0074	[0.0073, 0.0074]
Segregation × education (less than high school)	− 0.0117	[− 0.0118, − 0.0116]	− 0.0146	[− 0.0147, − 0.0146]	0.0198	[0.0197, 0.0198]
Segregation × education (high school graduate)	− 0.0006	[− 0.0006, − 0.0005]	0.0185	[0.0185, 0.0185]	0.0062	[0.0061, 0.0062]
Segregation × education (some college)	0.0047	[0.0046, 0.0047]	0.0039	[0.0039, 0.0039]	0.0131	[0.0130, 0.0131]

Note: Segregation was measured on a continuous scale (higher values representing higher racial segregation). The reference groups for the variables, “race,” “income,” and “education” were “White,” “ ≥ $100,000,” and “college graduate or more,” respectively

The results from the subgroup analysis of associations by metropolitan vs. non-metropolitan residential location are presented in Table 4. In metropolitan areas, informal caregivers in higher segregation were less likely to see a healthcare professional [*β* =  − 1.626; Wald 95%CI: − 1.636, − 1.617] (moderate); [*β* =  − 2.045; Wald 95%CI: − 2.054, − 2.035] (high), or use health services frequently [*β* =  − 0.743; Wald 95%CI: − 0.747, − 0.739] (moderate); [*β* =  − 0.5121; Wald 95%CI: − 0.516, − 0.508] (high)], but more likely to perceive a higher quality of care received [β = 0.662; Wald 95%CI 0.657, 0.667] (moderate); [*β* = 0.968; Wald 95%CI 0.963, 0.973] (high), when compared with those residing in low segregation areas. The results were different in non-metropolitan areas. Compared with those in low segregation, informal caregivers in a moderate level of segregation were less likely to see a healthcare professional [*β* =  − 27.286; Wald 95%CI: − 27.286, − 27.286] but were more likely to use health services frequently [*β* = 0.566; Wald 95%CI: 0.562, 0.571] (moderate); [*β* = 1.008; Wald 95%CI: 1.002, 1.013] (high), and more likely to perceive a higher quality of care received [*β* = 1.641; Wald 95%CI: 1.635, 1.647] (high).

**Table 4:** Subgroup analysis of associations by metropolitan residential location

	Metropolitan						Non-metropolitan					
	Healthcare visit		Frequency of healthcare visits		Quality of healthcare received		Healthcare visit		Frequency of healthcare visits		Quality of healthcare received	
	Estimate	Wald 95% CL	Estimate	Wald 95% CL	Estimate	Wald 95% CL	Estimate	Wald 95% CL	Estimate	Wald 95% CL	Estimate	Wald 95% CL
Segregation level (ref.: low)												
Moderate	− 1.626	[− 1.636, − 1.617]	− 0.743	[− 0.747, − 0.739]	0.662	[0.657, 0.667]	− 27.286	[− 27.286, − 27.286]	0.566	[0.562, 0.571]	− 24.906	[− 1119, 1069]
High	− 2.045	[− 2.054, − 2.035]	− 0.512	[− 0.516, − 0.508]	0.968	[0.963, 0.973]	**–**	**–**	1.008	[1.002, 1.013]	1.641	[1.635, 1.647]

Segregation level was measured on a categorical scale, “i.e., low, moderate, and high”

## Discussion

To our knowledge, this study is the first to examine associations between residential segregation, healthcare visits, frequency of healthcare visits, and perceived quality of care received among informal caregivers in the US. Overall, our findings suggest that informal caregivers living in highly or moderately segregated areas have fewer healthcare visits, less frequent healthcare visits, and lower perceived quality healthcare compared to those living in areas with lower residential segregation. Importantly, our results showed a dose–response relationship in most instances, indicating that as racial residential segregation increased, informal caregivers had a lower likelihood of having healthcare visits, a lower visit frequency, and a poorer perception of the quality of healthcare received. Further, caregivers who were members of some mineralized populations (i.e., Blacks) and those with critically lower socioeconomic status (i.e., income of US$ < $35,000 and less than high school in education) had a lower likelihood of having healthcare visits and lower visit frequencies with increased levels of residential segregation. The latter subgroup analyses for subcategories of caregivers revealed that, generally, there was a positive sentiment of quality care received as levels of residential segregation increased, except for Black caregivers and those residing in moderately segregated non-metropolitan locations.

Our results, overall, are consistent with a few findings reported in the literature [61–64]. For instance, Munir et al. [64] examined the association of residential segregation with the diagnosis, treatment, and outcomes among patients with hepatopancreatic biliary (HPB) cancer. Results from their analysis showed that Black individuals living in highly segregated areas were less likely to be diagnosed early with HPB cancer or receive timely treatment, which in turn led to a higher risk of mortality compared to White patients residing in less segregated areas. Moreover, several other studies highlighted direct associations between segregation and access to quality healthcare services and providers. Their findings indicate that minorities living in highly segregated areas experienced reduced access to care, had fewer qualified providers, fewer referrals to other medical services, and had higher rates of unmet healthcare needs compared to Whites [65–70]. These findings suggest that higher residential segregation levels are associated with lower access to or utilization of needed healthcare, especially for individuals with a minority background, and corroborate our results for informal caregiver populations.

Several factors may account for the lower healthcare visits and poor perceived quality of care observed among the larger pool of informal caregivers living in highly segregated neighborhoods in the current study. First, the intense burden of caregiving, which includes physical, mental, and emotional stress, may limit caregivers’ ability to access healthcare when necessary [4, 71, 72]. Second, residing in a segregated community could significantly restrict access to healthcare services [43]. Low-income segregated areas often experience the closure of essential public healthcare facilities, become medication deserts, and face shortages of primary care physicians. These have substantial adverse consequences, specifically for individuals of minority racial and ethnic groups and those with lower socioeconomic status who are more likely to reside in more segregated areas. For example, Eberth et al. [73] investigated healthcare accessibility across different geographic regions, and their results showed that Black or American Indian/Alaska Native communities in rural areas were significantly further from hospitals providing emergency services, trauma care, obstetrics, outpatient surgery, intensive care, and cardiac care compared to the White population. Other studies show that communities with a higher proportion of African American and Latino populations are four times more likely than non-Latino White communities to experience a shortage of specialists, irrespective of the community’s income level [74], and locations with a higher percentage of the Black population are associated with a higher likelihood of ED closure [75]. This phenomenon is termed “White flight” in healthcare, which is similar to “White flight” in neighborhoods. It refers to providers and hospitals relocating to more affluent suburban areas primarily occupied by White populations [70]. Third, segregated areas are often characterized by restricted educational and employment opportunities, leading to concentrated poverty [76], the absence of resources that facilitate access to care, such as public transportation, leading to longer commute times, and limited car ownership, which might further discourage informal caregivers from seeking healthcare services.

Our findings also suggest that as racial segregation increased, there was a direct association between caregivers’ educational level and the probability of having a healthcare visit. Caregivers with lower educational attainment residing in higher segregated locations were less likely to have healthcare visits. This finding highlights the impact of structural barriers and social risk factors like lower educational levels on healthcare visits, and it is consistent with findings from published literature. Surprisingly, certain subgroups of caregivers living in higher segregated areas, except for caregivers identified as Blacks, reported a better perception of healthcare quality. This is unexpected and contradicts existing literature linking residence in segregated areas and caregiving roles to lower perceived quality of care and adverse health outcomes, such as an elevated risk of cardio-metabolic diseases like hypertension, heart disease, obesity, and stroke [77]. Plausible interpretations include that perceived improvements in healthcare quality may not necessarily align with the actual receipt of high-quality care. Since the assessment of healthcare quality is subjective, people might normalize lower quality care and consequently report a higher perception of care quality [43]. Moreover, as highlighted by Caldwell et al., [43], the perceived higher quality of care among individuals in segregated areas may not necessarily translate into access to or receipt of quality specialist services, which can be challenging to obtain in highly segregated communities. Another plausible explanation for this finding in the current study is having a relatively smaller sample for analysis, thus exploring associations between residential segregation and perceived, or actual, quality healthcare services warrants further investigation.

This study has several limitations, and the findings need to be interpreted in light of those limitations. First, the data obtained from the HINTS and the HDLP 2020 project are cross-sectional in nature, and causality cannot be inferred given that the direction of associations can be difficult to interpret. Second, our analytical investigations might be missing important unmeasured confounding factors that could potentially influence the computed results. Such factors might have a correlation with residential segregation, informal caregiving responsibility, healthcare visits, and individuals’ perceptions of quality healthcare. Therefore, we were bound by the information provided and the variables available in the dataset. Third, survey respondent-related and data management bias might have been introduced during the process of data collection and administration, respectively. For example, respondents are sometimes liable to have information and recall bias and might not accurately recall conditions and events such as the frequency of healthcare visits. Further, we could not confirm whether the types of healthcare visits were discretionary or non-discretionary. Whether seeing a healthcare provider was during hospital admission or for preventive or screening purposes, making these distinctions would have resulted in differential impacts of segregation on access to essential and non-essential healthcare services, with more specific implications for healthcare policy and practice. Lastly, given that only close to 16% of caregivers had a non-metropolitan residential location, the metro and non-metro representation of caregivers is not equally distributed; thus, the main findings as presented in Table 2 and their interpretations could potentially pertain only to segregation in metropolitan areas. These limitations provide an opportunity for further studies on this topic, and future research could expand upon these findings by potentially designing and conducting prospective, longitudinal studies to strengthen evidence and establish consensus on these associations in the caregiving field.

## Conclusion

In summary, the main findings of the present study highlight that greater levels of residential segregation were associated with a decrease in healthcare visits, reduced visit frequency, and a poorer perception of healthcare quality among informal caregivers. Certain groups of minority backgrounds, specifically Black caregivers and those of lower socioeconomic status, were more likely to be impacted in terms of healthcare access and utilization when residing in areas with higher residential segregation. These results underscore the critical role of community-level and contextual factors, such as historical segregation and the built environment, that potentially influence access to and utilization of quality health services among informal caregivers. Further, despite the need for further studies to consolidate and support the findings presented in the current study, our findings serve as relevant evidence for health policymakers, emphasizing the dual inequalities faced by informal caregivers residing in highly segregated areas in the US. Not only do they struggle with the challenges of caregiving, but they also contend with the additional burden of residing in segregated areas and face challenges associated with geographic barriers related to access to quality healthcare, which further constrain their access to care and exacerbate adverse outcomes. This again underscores the pressing need for tailored programs and targeted policy interventions to address the unique challenges confronted by informal caregivers in low-income, segregated communities, aiming to alleviate both the caregiving burden and the impact of residential segregation on healthcare disparities. Scholars and other researchers interested in further exploring the impact of segregation on access and quality of healthcare services may plan for designing and implementing mixed methods or prospective-longitudinal studies and account for further individual and contextual factors shaping the associations. Meanwhile, plans for future programs aimed at promoting health and well-being of informal caregivers may need to account for both individual- and community-level caregiving factors in an effort to improve access to quality healthcare and reduce disparities in access and utilization of healthcare services among informal caregivers.

## Data Availability

Restricted data were made available to the authors by the National Cancer Institute upon data request and study proposal approval. Other utilized data in this study can be accessed and downloaded at: https://hints.cancer.gov/data/Default.aspx.
